# Comment on “Application of PK/PD Modeling in Veterinary Field: Dose Optimization and Drug Resistance Prediction”

**DOI:** 10.1155/2017/7698452

**Published:** 2017-07-18

**Authors:** Anna Benini, Guido Francesco Fumagalli

**Affiliations:** Department of Diagnostics and Public Health, Pharmacology Section, University of Verona, Verona, Italy

While performing a literature search in PubMed on application of PK/PD parameters in veterinary, we found the review “Application of PK/PD Modeling in Veterinary Field: Dose Optimization and Drug Resistance Prediction” by Ahmad et al. published in BioMed Research International [1]. This review deals with antimicrobial drugs used in veterinary and with pharmacokinetic/pharmacodynamic modeling developed to maximize drug efficacy, establish appropriate dosage regimens, and reduce side effects.

Although the review is interesting and well-articulated, we disagree with the content of Table 3.

In that table (page 5) the different classes of antimicrobial drugs are divided into two groups. For group 1 the common criterion is the AUC/MIC ratio (*C*_max_/MIC or AUC_0–24_/MIC). For group 2, %*T*_>MIC_ is the classification criterion. The table is indeed inspired by a previous publication by Martinez et al. published in 2012 [2]. The grouping of antibiotics based on PK/PD parameters is also discussed by Martinez et al. in 2014 [3], by Ambrose et al. in 2007 [4], and by several other authors.

In all cases Ketolides, Lincosamides (clindamycin), and Glycopeptides (Vancomycin) are identified by the AUC/MIC ratio as PK/PD reference parameters. They should therefore be included in group 1 and not in group 2 as done by the authors of the review published in your journal.

Those antibiotics have effects that are time dependent but this is not a sufficient criterion to apply as PK/PD criterion %*T*_>MIC_. Indeed, antimicrobial efficacy of Vancomycin, Clindamycin, and Telithromycin is related to *C*_max_/MIC or AUC_0–24_/MIC.

It is well known that antimicrobial agents that interact with the cell wall usually display time-dependent bactericidal activity that correlates with %*T*_>MIC_. On the contrary, agents that inhibit protein synthesis have a concentration-dependent bactericidal action which correlates well with the AUC/MIC ratio. Thus, Ketolides and Lincosamides are usually included in group 1 and not 2 (see [2, 3]).

However it should be noted that also agent acting on the bacterial wall may be included in group 1 (bactericidal action correlating more with the AUC/MIC ratio than with the %*T*_>MIC_). In the case of Vancomycin, whose half-life and prolonged PAE fully satisfy the condition time-dependent bactericidal effect, the real predictor of efficacy is more closely related to plasma concentration [5, 6].

The implication of including an agent in group 1 or 2 is definition of the therapy in terms of dose and pharmacokinetics. Including one agent in one group instead of the other is not trivial and may lead to therapeutic errors. We therefore strongly recommend that the organization of Table 3 is revised according to our observation and to the abundant literature.
